# A study of the top-cited studies on drug therapy for HIV

**DOI:** 10.3389/fphar.2022.1007491

**Published:** 2022-08-31

**Authors:** Jie Tang, Yanwen Yuan, Lei Sun, Bo Wu, Lin Yu

**Affiliations:** ^1^ Editorial Board of Journal of Sichuan University (Medical Science Edition), Sichuan University, Chengdu, China; ^2^ West China School of Public Health and West China Fourth Hospital, Sichuan University, Chengdu, China

**Keywords:** top-cited, HIV, drug therapy, citation analysis, hotspots

## Abstract

**Background** Research on drug therapy for HIV remained major hot-spots, but relevant data were not satisfactory. We aimed to assess the status and trends of the most cited studies on drug therapy for HIV by using bibliometric methods.

**Methods** The Web of Science Core Collection database was searched for the drug therapy for HIV studies. The period for retrieval was from the beginning of the database to July 26, 2022. The 100 top cited studies were selected. These general information and bibliometric data were collected and analyzed. VOS viewer software was used for visualization analysis.

**Results** The number of citations for the 100 top cited studies ranged from 451 to 5597 and were published from 1987 to 2017. These studies were published in 29 journals. The top 3 journals in terms of the number of studies were the New England Journal of Medicine (*n* = 22), Lancet (*n* = 15), and JAMA (*n* = 13). The most frequently nominated author was Matthias Eiger from the University of Bern, who has contributed 5 studies. United States, Switzerland, and England contributed most of the highly cited studies. Research hot spots reflected clinical trials, treatment adverse events, basic research, and clinical adherence.

**Conclusion** The majority of 100 top-cited studies have been published in the United States, and primarily focused on treatment adverse events, basic research, and clinical adherence. They provide a basic list of the most important and influential academic contributions to literature of HIV drug treatment for researchers.

## Introduction

Human immunodeficiency virus (HIV) was a global health problem, which affects people of all ages and continues to threaten social and economic development (Lazarus et al., 2021; Annor et al., 2022). At the end of 2020, an estimated 37.7 million people worldwide were infected with HIV/AIDS, of which two-thirds came from Africa (Shahzad et al., 2022b). In order to control this disease, it was very important to diagnose the disease early, start the treatment early, and keep the treatment persistence of the virus load suppressed (Shahriar et al., 2021; Annor et al., 2022). Free highly active antiretroviral therapy (HAART) was very important (Bani et al., 2020; Gopalakrishnan et al., 2020; Mai et al., 2020; Getaneh et al., 2021). The introduction of HAART effectively reduced the burden of HIV (Bani et al., 2020; Getaneh et al., 2021). The outcome of drug therapy for HIV mainly depended on the application of antiretroviral drugs (Mayer et al., 2022). Demands for antiretroviral drugs were very high, and the researches on new drugs were also very important (de Gaetano Donati et al., 2004; Dore and Cooper, 2006). Knowing the research trends of the drug therapy for HIV was very important for future works (de Gaetano Donati et al., 2004), and there was no such study up to now. Using the bibliometrics method might be a potential method (Jenkins et al., 2022; Ong et al., 2022).

Bibliometrics is a method of quantitative science to analyze research publications (Paschoal et al., 2022; Zhao et al., 2022), and increased markedly concomitant with the sharp expansion of literature (Paschoal et al., 2022; Zhao et al., 2022). Citation analysis was an important methodology frequently used and allowed analysis of the seminal studies (Paschoal et al., 2022; Shahzad et al., 2022a), outstanding institutes or scientists, progressions, and the emerging trends in a specific field (Jenkins et al., 2022; Li et al., 2022; Ong et al., 2022). Analyzing the top cited studies could help a specific field (Sarker et al., 2022). Up to now, there was no study analyzing the 100 top cited studies of drug therapy for HIV. Thus, we performed the current study.

## Materials and methods

### Data sources

The most cited drug therapy for HIV studies was retrieved from the Web of Science Core Collection from the beginning of the database to April 10, 2022 (updated to July 26, 2022), in Sichuan University, Chengdu, China. HIV, drug therapy, and antiviral were used as search terms. Studies were sorted in descending order according to the number of times WOS cited (Zhao et al., 2022), and the 100 most cited studies were included.

### Data extraction

Two independent researchers evaluated each identified study to ensure that the studies involved drug therapy for HIV (Sarker et al., 2022; Shahzad et al., 2022a), regardless of the article type. The following information was collected from the included studies (Ong et al., 2022; Sarker et al., 2022; Shahzad et al., 2022a): authors, year of publication, title, journal, article type, author’s affiliations, country, and citation frequency.

### Statistical analysis

Microsoft excel 2007 software was used for descriptive statistical analysis of authors, institutions, countries, journals, and the number of citations (Shahzad et al., 2022a). VOS viewer 1.6.18 software was used for visual analysis (Zhao et al., 2022), a mapping network diagram of keyword co-occurrences, and co-authored researchers (Paschoal et al., 2022; Zhao et al., 2022). In the terms’ map, each circle represented a term. The size of the circle indicated how often it appeared (Yang et al., 2022). The color of a circle indicated the average number of citations received (Yang et al., 2022). The term map visualization appears at least five times in the 100 studies (Paschoal et al., 2022; Zhao et al., 2022).

## Results

### Publications and citations

All of the 100 top cited studies were written in English (Supplementary Table S1). The citations ranged from 451 to 5597 times by the time we retrieved them, with a median citation of 652 and an average citation of 908. The total number of citations was 90,814. The top 100 top-cited studies were published from the years 1987–2017. It was worthy to mention the top 3 studies. The first study was named Antiretroviral Therapy for the Prevention of HIV-1 Transmission, which was published in the New England Journal of Medicine in 2016 and had also been cited 5597 times (Cohen et al., 2016). The second top cited paper was named “Prevention of HIV-1 Infection with Early Antiretroviral Therapy” (Cohen et al., 2011), which was published in the New England Journal of Medicine in 2011 and has also been cited 5597 times. The third top-cited study named “Reduction of Maternal-Infant Transmission of Human-Immunodeficiency-Virus Type-1 With Zidovudine Treatment” (Connor et al., 1994), was published in the New England Journal of Medicine in 1994, which had been cited 2773 times.

### Countries and institutions

When analyzing the counties by based on the corresponding author, a total of 10 countries contributed to the 100 top cited studies related to drug therapy for HIV(Table 1). The majority of these countries contributed more than 2 studies. The United States dominated the drug therapy for HIV field, contributing 68 of the top 100 studies, with 66,294 citations and an average citation of 974. Switzerland ranked second, contributing 11 studies, with 8344 citations and an average citation of 758. Next, England contributed 6 studies, with 4932 citations and an average citation of 822. France contributed 5 studies, with 3869 citations and an average citation of 773. Studies from Denmark had the highest average citation (1264).

**TABLE 1:** Countries for the top 100 studies in drug therapy for the HIV field.

Country	Number of studies	Number of citations	Average of citation
United States	68	66,294	974
Switzerland	11	8344	758
England	6	4932	822
France	5	3869	773
Canada	4	2318	579
Denmark	2	2529	1264
Australia	1	464	464
Italy	1	492	492
Netherlands	1	600	600
South Africa	1	972	972

As for the institutions, a total of 25 institutions contributed more than 1 top-cited study related to drug therapy for HIV. In Table 2, the majority of these institutions were from the United States, only 7 were from other countries. The University of Bern in Switzerland and the University of North Carolina in the United States both had the highest contributions as an institution, with 5 studies. While four institutions (Brown University, Johns Hopkins University, University of California San Diego, and The National Institute of Allergy and Infectious Diseases) from the United States contributed four studies.

**TABLE 2:** Institutions for the top 100 studies in drug therapy for HIV field.

Institutions	Country	Number of study
Univ British Columbia	Canada	2
Toronto Gen Hosp	Canada	2
Univ Bristol	England	2
HOP LA PITIE SALPETRIERE	France	2
Univ Bern	Switzerland	5
CHU Vaudois	Switzerland	2
WHO	Switzerland	2
AIDS Res Consortium Atlanta	United States	2
Boston Univ	United States	2
Brown Univ	United States	4
Columbia Univ Coll Phys and Surg	United States	3
Johns Hopkins Univ	United States	4
Massachusetts Gen Hosp	United States	2
Merck Res Labs	United States	2
NCI	United States	2
NIAID	United States	4
Northwestern Univ	United States	2
Rockefeller Univ	United States	2
San Francisco Gen Hosp	United States	2
Univ Calif San Diego	United States	4
Univ Calif San Francisco	United States	2
UNIV MIAMI	United States	2
Univ N Carolina	United States	5
Univ Pittsburgh	United States	2
Univ Washington	United States	3

### Publishing years

These studies were published between 1987 and 2017, with the largest number of studies published in 1997 (*n* = 10). Table 3 showed the number of studies published in each 5-years interval. Only 7 studies were published before 1990, while 93 were published after 1990. The largest number of studies published in a single interval was 37, which occurred in 1996–2000. For the citations, the total 5-years interval citations of the article in 1996–2000 ranked first, with 31,607 citations, while the average citations in 2016–2020 ranked first, with 2261 citations.

**TABLE 3:** 5-years scientific productions and citations.

Publishing year	Number of studies	Total citations	Mean citations
1986–1990	7	8176	1168
1991–1995	5	5652	1130
1996–2000	37	31,607	854
2001–2005	16	10,091	630
2006–2010	22	15,875	721
2011–2015	10	12,630	1263
2016–2020	3	6783	2261

### Authors

The authors who contributed to more than 1 study as the first author or corresponding author are shown in Table 4. A total of 13 authors contributed more than 1 paper as the first author17 and authors contributed more than 1 paper as corresponding authors. Among the first authors, Charles C J Carpenter from Brown University in the United States contributed four studies and ranked first, almost all high productivity first authors were from the United States, and only 1 was from Switzerland1 and from Canada. Among the corresponding authors, Matthias Egger from the University of Bern contributed 5 studies and ranked first12; high productivity corresponding authors were from the United States, and only 2 from Switzerland1, from France2, and from Canada.

**TABLE 4:** Authors with more than 1 paper as first author or corresponding author included in the top-100 studies.

Author		Number of studies	Institution	Country
Corresponding author	Egger, M	5	Univ Bern	Switzerland
	Carpenter, CCJ	4	Brown Univ	USA
	Hammer, SM	3	Columbia Univ Coll Phys & Surg	USA
	Cohen, MS	3	Univ N Carolina,	USA
	Walker, BD	2	Univ Calif San Francisco	USA
	Thompson, MA	2	Aids Res Consortium Atlanta	USA
	Telenti, A	2	Univ Lausanne Hosp	Switzerland
	Siliciano, RF	2	Johns Hopkins Univ	USA
	Richman, DD	2	Univ Calif San Diego	USA
	Montaner, JSG	2	Univ British Columbia	Canada
	Mitsuya, H	2	NCI	USA
	Hunt, PW	2	San Francisco Gen Hosp	USA
	Ho, DD	2	Rockefeller Univ	USA
	Heaton, RK	2	Univ Calif San Diego	USA
	Fischl, MA	2	Univ Miami	USA
	Autran, B	2	Hop La Pitie Salpetriere	France
	Walmsley, SL	2	Toronto Gen Hosp	Canada
First author	Carpenter, CCJ	4	Brown Univ	USA
	Cohen, MS	3	Univ N Carolina,	USA
	Deeks, SG	2	San Francisco Gen Hosp,	USA
	Egger, M	2	Univ Bern	Switzerland
	Finzi, D	2	Johns Hopkins Univ	USA
	Fischl, MA	2	Univ Miami	USA
	Hammer, SM	3	Columbia Univ Coll Phys & Surg	USA
	Heaton, RK	2	Univ Calif San Diego	USA
	Hunt, PW	2	San Francisco Gen Hosp	USA
	Mitsuya, H	2	NCI	USA
	Richman, DD	2	Univ Calif San Diego	USA
	Thompson, MA	2	Aids Res Consortium Atlanta	USA
	Walmsley, SL	2	Toronto Gen Hosp	Canada

### Journals

Table 5 listed the relevant journal of 100 top cited studies, according to the included studies, we ranked the published journals in this field in descending order, and a total of 29 journals were included. The *New England Journal of Medicine* journal published most of the studies (*n* = 22), with 30,702 citations, followed by *Lancet*, which published 15 studies with 11,125 citations, and the *JAMA* published 13 studies with 8786 citations. According to the JCR report released in 2022, the journal with the highest impact factor was *Lancet*, with an impact factor of 202.731. Most journals were from the United States and only four were from England. As for the category quartile ranking, 24 journals were in Q1, 2 were in Q2 and 3 were in Q3. Most journals were journals associated with infectious diseases or virology, while a few journals were top general journals.

**TABLE 5:** Journal distribution of 100 top cited studies in drug therapy for HIV field.

Journal	Number of studies	Total citation	Country	Impact factor	Category quartile
New England Journal of Medicine	22	30,702	United States	176.079	Q1
Lancet	15	11,125	England	202.731	Q1
JAMA-Journal of The American Medical Association	13	8786	United States	157.335	Q1
Nature	6	5600	England	69.504	Q1
Science	6	7475	United States	63.798	Q1
Aids	5	3327	United States	4.632	Q2
Journal of Infectious Diseases	3	1625	United States	7.759	Q1
Nature Medicine	3	2700	United States	87.241	Q1
Annals of Internal Medicine	2	3021	United States	51.598	Q1
Clinical Infectious Diseases	2	970	United States	20.999	Q1
Jaids-Journal of Acquired Immune Deficiency Syndromes	2	1511	United States	3.771	Q3
Jnci-Journal of The National Cancer Institute	2	1120	United States	11.816	Q1
Journal of Virology	2	1169	United States	6.549	Q2
Proceedings of The National Academy of Sciences of The United States of America	2	2079	United States	12.779	Q1
Aids and Behavior	1	463	United States	4.852	Q1
Archives of Internal Medicine (Jama Internal Medicine)	1	562	United States	44.409	Q1
Bmj-British Medical Journal	1	524	England	93.467	Q1
Health Psychology	1	513	United States	5.556	Q1
Immunity	1	531	United States	43.473	Q1
Journal of Acquired Immune Deficiency Syndromes	1	718	United States	3.586	Q1
Journal of Medicinal Chemistry	1	492	United States	8.039	Q1
Journal of Neurovirology	1	1009	United States	3.739	Q3
Journal of Pharmacology and Experimental Therapeutics	1	482	United States	4.441	Q2
Journal of The International Aids Society	1	541	England	6.707	Q1
Lancet HIV	1	486	England	16.07	Q1
Lancet Infectious Diseases	1	471	United States	71.421	Q1
Neurology	1	1560	United States	11.8	Q1
Plos Medicine	1	553	United States	11.613	Q1
Plos Pathogens	1	699	United States	7.464	Q1

### Cooperation

**FIGURE 1:**
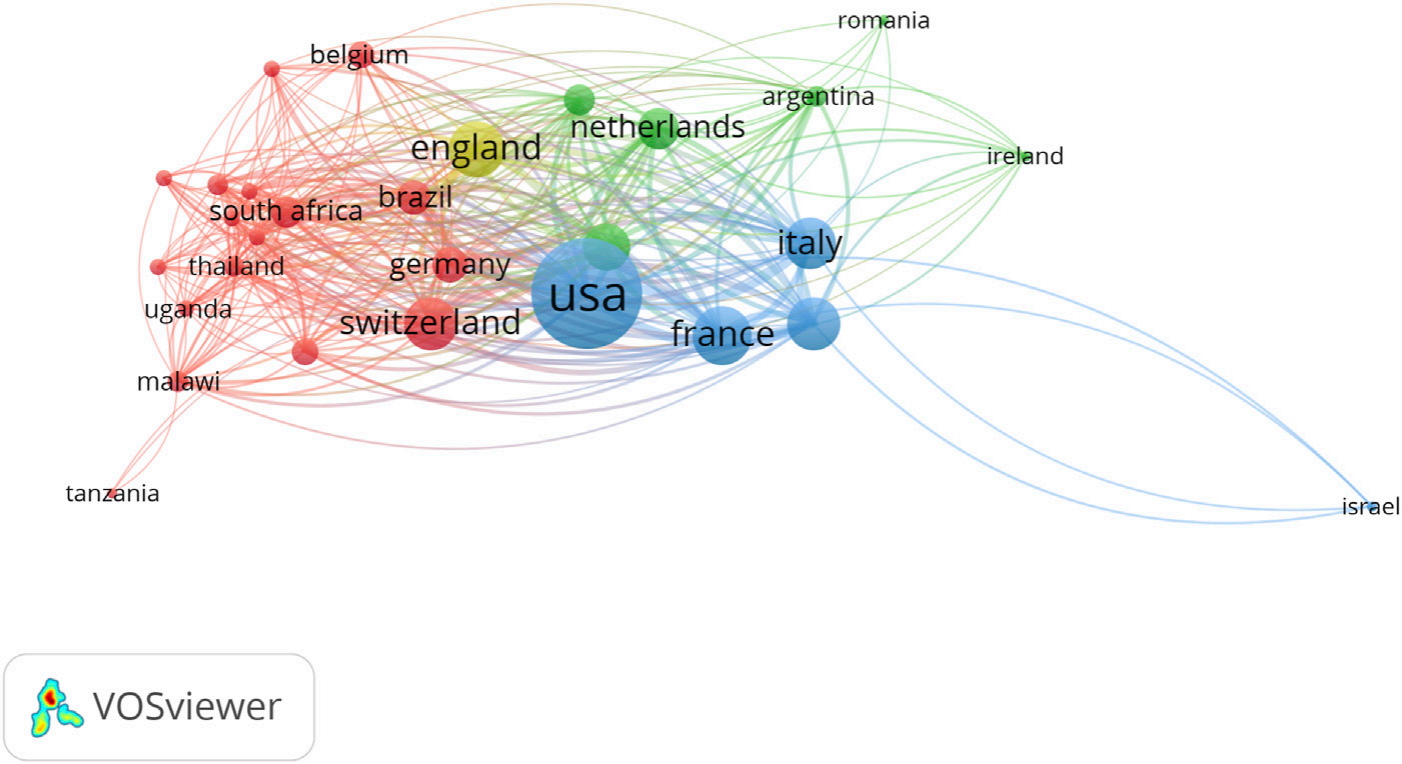
Cooperation of different countries.

The cooperation among different countries was shown in Figure 1. We could find that there were 3 mainly cooperation networks, the first is the United States, which included the United States, France, and Italy (Blue in the figure), and the second cooperation network included counties in European countries, including, Switzerland, Germany, South Africa, Uganda, Malawi; the third cooperation includes Netherlands, Argentina, Romania, and Ireland. When analyzing the cooperation based on institutions (Supplementary Figure S1), almost all cooperation was based on institutions in the United States. When analyzing the cooperation based on authors, almost all cooperation was based on the United States(Supplementary Figure S2).

### Keywords and research hotspots

**FIGURE 2:**
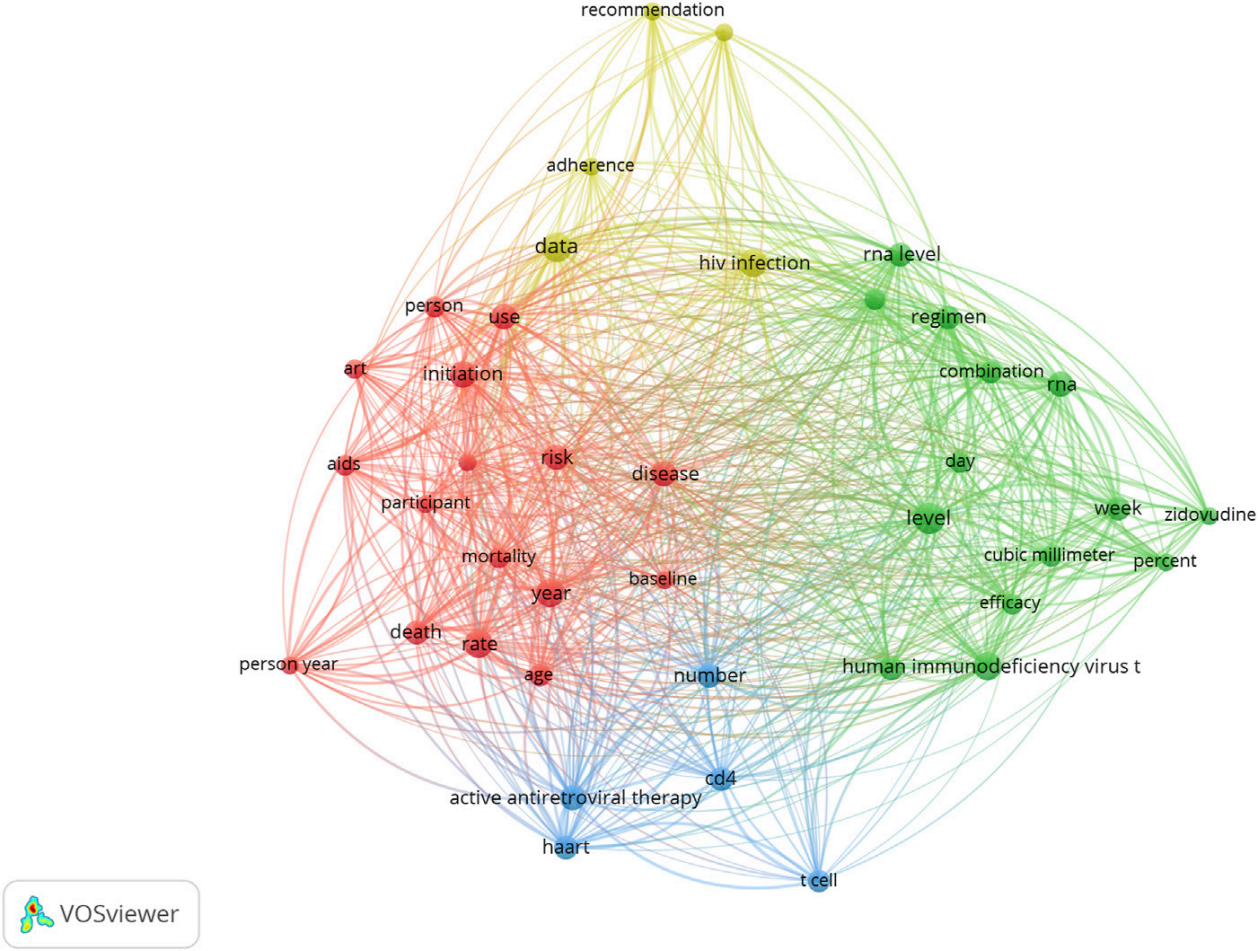
Research hotspots of drug therapy for HIV.

The keyword co-occurrence network of the 100 most cited studies is shown in Figure 2. The top keywords included level, data, HIV infection, rate, year, human immunodeficiency virus type, initiation, risk, use, and active antiretroviral therapy. Accordingly, the terms or phrases associated with drug therapy are divided into four clusters, represented by four colors (red, green, blue, and yellow). As shown in Figure 2, many terms might reflect clinical trials, treatment adverse events, basic research, and clinical adherence.

## Discussion

The current bibliometric study highlighted the characteristics of the 100 cited studies on drug therapy for HIV from the Web of Science Core Collection database. The analysis showed the top cited publication’s growth on drug therapy for HIV research. The cumulative number of published studies showed increased interest in therapy efficacy, drug adverse event, and the mechanism of novel drugs for HIV, which provided a picture of the future of drug therapy for HIV. These studies were published between 1987 and 2017. Most studies focused on the therapeutic efficacy, and adverse events.

The top 1 most cited study was published in 2016 (Cohen et al., 2016), entitled Antiretroviral Therapy for the Prevention of HIV-1 Transmission, with 5597 citations. This study provided insight into more than 5 years of follow-up to assess the durability of antiretroviral therapy (ART) for the prevention of HIV-1 transmission, which suggested that early initiation of ART led to a sustained decrease in genetically linked HIV-1 infections in sexual partners. The top 2 most cited study was published in 2011 (Cohen et al., 2011), entitled Prevention of HIV-1 Infection with Early Antiretroviral Therapy, with 5597 citations. This paper provided insight into Antiretroviral therapy for the transmission of human immunodeficiency virus type 1 (HIV-1) in serodiscordant couples. The top 3 most cited study was published in 1994 (Connor et al., 1994), entitled Reduction of Maternal-Infant Transmission of Human-Immunodeficiency-Virus Type-1 With Zidovudine Treatment, with 2773 citations. This study found the results that pregnant women with mildly symptomatic HIV disease and no prior treatment with antiretroviral drugs during the pregnancy, a regimen consisting of zidovudine given antepartum and intrapartum to the mother and the newborn for 6 weeks reduced the risk of maternal-infant HIV transmission by approximately two thirds. What’s more, from the analysis of the most frequently occurring keyword we can learn that keywords in this field are deep and tight (Ong et al., 2022; Shahzad et al., 2022a), and these topics have always been the focus of research.

In the study, the involved 100 studies were published in 29 journals, with an impact factor range of 3.771–202.731. It was found that the journals published studies were of high quality (Shahzad et al., 2022a), 82.76% of the journals were categorized in Q1 quartile, 6.90% were in Q2 quartile and 10.34% were in Q3 quartile. 51% of the most cited studies were published in the top four general journals, including New England Journal of Medicine (IF = 176.079), followed by Lancet (IF = 202.731), JAMA (IF = 157.335), and BMJ (IF = 93.467). It was not surprising that researchers preferred to submit high-quality studies to journals with high impact factors (Sarker et al., 2022; Zhao et al., 2022), these results indicated that the field of drug therapy for HIV was very mature and has attracted much attention from researchers (Staszewski et al., 1999; Carpenter et al., 2000; Paterson et al., 2000).

We identified the countries and institutions’ distribution of these publications and found that the United States had the largest number of publications, followed by Switzerland. The total number of studies published in these countries was nearly 80% of the top 100 studies. The University of Bern in Switzerland and the University of North Carolina in United States were the top 2 institutions that publish the most top studies, each contributing 5 studies. A total of 18 institutions contributed more than 2 top cited studies; which suggested that United States was still at the forefront of the world in the field of drug therapy for HIV. Experts all over the world participated in the current field, especially in North America and Europe. Professor Matthias Egger from the University of Bern in Switzerland published the most studies (5 publications). Scholars from Africa and South America seemed not published top cited studies on drug therapy for HIV.

From the cooperation analysis, authors from institutions in the United States contributed the most impact cooperation for the top studies (Richman et al., 1987; Hammer et al., 1996; Chun et al., 1997), while this cooperation also involved some African countries. These findings could be attributed to an enhanced opportunity for a general understanding of drug therapy for HIV for new readers about the natural history and trends within the drug therapy for HIV field. In addition, more creative and revolutionary ideas and worldwide cooperation efforts among academics, drug developers and governmental funding bodies must be encouraged and promoted, and then provide more drugs to therapy the disease. Although drug therapy of HIV is highly effective, eradication of virus has not been possible to date, future new researches may focus on eradication of the virus by using long-acting antiviral agents, low side effect agents, such as combination anti-HIV antibodies (Sneller et al., 2022), which will help individuals with HIV.

There were some limitations in the analysis. First, as publications are filtered according to citations number, publications in recent years might be ignored because they might not be cited so many times (Jenkins et al., 2022; Ong et al., 2022). Second, the current study did not provide further analysis based on such as reviews, meta-analyses, and clinical guidelines or recommendations (Li et al., 2022). Third, we only included publications in English recorded on the Web of Science Core Collection (Shahzad et al., 2022a), and language limitations might occur, which cannot guarantee the acquisition of comprehensive HIV treatment literature.

In conclusion, this current study highlights the 100 top cited studies in drug therapy for HIV. The United States is the most productivity country. Treatment adverse events, basic research, and clinical adherence are the main hot spots. Our results provide support for future research for developing new drugs for HIV.

## Data Availability

The original contributions presented in the study are included in the article/Supplementary Material, further inquiries can be directed to the corresponding author.
