# Supplementary material for: A study of the top-cited studies on drug therapy for HIV
Source: Front Pharmacol. 2022 Aug 31;13:1007491. doi: 10.3389/fphar.2022.1007491 (PMC9473148; doi:10.3389/fphar.2022.1007491)

**Supplement table 1 The included 100 top cited studies**

| Ranking | Title | Journal | Total citation | Year |
| --- | --- | --- | --- | --- |
| 1 | Antiretroviral Therapy for the Prevention of HIV-1 Transmission | NEW ENGLAND JOURNAL OF MEDICINE | 5597 | 2016 |
| 2 | Prevention of HIV-1 Infection with Early Antiretroviral Therapy | NEW ENGLAND JOURNAL OF MEDICINE | 5597 | 2011 |
| 3 | REDUCTION OF MATERNAL-INFANT TRANSMISSION OF HUMAN-IMMUNODEFICIENCY-VIRUS TYPE-1 WITH ZIDOVUDINE TREATMENT | NEW ENGLAND JOURNAL OF MEDICINE | 2773 | 1994 |
| 4 | THE EFFICACY OF AZIDOTHYMIDINE (AZT) IN THE TREATMENT OF PATIENTS WITH AIDS AND AIDS-RELATED COMPLEX - A DOUBLE-BLIND, PLACEBO-CONTROLLED TRIAL | NEW ENGLAND JOURNAL OF MEDICINE | 2394 | 1987 |
| 5 | Adherence to protease inhibitor therapy and outcomes in patients with HIV infection | ANNALS OF INTERNAL MEDICINE | 2370 | 2000 |
| 6 | Identification of a reservoir for HIV-1 in patients on highly active antiretroviral therapy | SCIENCE | 2330 | 1997 |
| 7 | Initiation of Antiretroviral Therapy in Early Asymptomatic HIV Infection | NEW ENGLAND JOURNAL OF MEDICINE | 1744 | 2015 |
| 8 | THE TOXICITY OF AZIDOTHYMIDINE (AZT) IN THE TREATMENT OF PATIENTS WITH AIDS AND AIDS-RELATED COMPLEX - A DOUBLE-BLIND, PLACEBO-CONTROLLED TRIAL | NEW ENGLAND JOURNAL OF MEDICINE | 1703 | 1987 |
| 9 | Latent infection of CD4(+) T cells provides a mechanism for lifelong persistence of HIV-1, even in patients on effective combination therapy | NATURE MEDICINE | 1627 | 1999 |
| 10 | HIV WITH REDUCED SENSITIVITY TO ZIDOVUDINE (AZT) ISOLATED DURING PROLONGED THERAPY | SCIENCE | 1584 | 1989 |
| 11 | HIV-associated neurocognitive disorders persist in the era of potent antiretroviral therapy CHARTER Study | NEUROLOGY | 1560 | 2010 |
| 12 | Treatment with indinavir, zidovudine, and lamivudine in adults with human immunodeficiency virus infection and prior antiretroviral therapy | NEW ENGLAND JOURNAL OF MEDICINE | 1524 | 1997 |
| 13 | Positive effects of combined antiretroviral therapy on CD4(+) T cell homeostasis and function in advanced HIV disease | SCIENCE | 1506 | 1997 |
| 14 | Presence of an inducible HIV-1 latent reservoir during highly active antiretroviral therapy | PROCEEDINGS OF THE NATIONAL ACADEMY OF SCIENCES OF THE UNITED STATES OF AMERICA | 1475 | 1997 |
| 15 | Decay characteristics of HIV-1-infected compartments during combination therapy | NATURE | 1474 | 1997 |
| 16 | Changes in thymic function with age and during the treatment of HIV infection | NATURE | 1444 | 1998 |
| 17 | Universal voluntary HIV testing with immediate antiretroviral therapy as a strategy for elimination of HIV transmission: a mathematical model | LANCET | 1406 | 2009 |
| 18 | Prognosis of HIV-1-infected patients starting highly active antiretroviral therapy: a collaborative analysis of prospective studies | LANCET | 1183 | 2002 |
| 19 | IMPACT OF IMPROVED TREATMENT OF SEXUALLY-TRANSMITTED DISEASES ON HIV-INFECTION IN RURAL TANZANIA - RANDOMIZED CONTROLLED TRIAL | LANCET | 1105 | 1995 |
| 20 | Mortality in the highly active antiretroviral therapy era - Changing causes of death and disease in the HIV outpatient study | JAIDS-JOURNAL OF ACQUIRED IMMUNE DEFICIENCY SYNDROMES | 1046 | 2006 |
| 21 | HIV-associated neurocognitive disorders before and during the era of combination antiretroviral therapy: differences in rates, nature, and predictors | JOURNAL OF NEUROVIROLOGY | 1009 | 2011 |
| 22 | Early Antiretroviral Therapy and Mortality among HIV-Infected Infants. | NEW ENGLAND JOURNAL OF MEDICINE | 972 | 2008 |
| 23 | Mortality of HIV-1-infected patients in the first year of antiretroviral therapy: comparison between low-income and high-income countries | LANCET | 897 | 2006 |
| 24 | Antiretroviral therapy in adults - Updated recommendations of the International AIDS Society-USA Panel | JAMA-JOURNAL OF THE AMERICAN MEDICAL ASSOCIATION | 849 | 2000 |
| 25 | Administration of vorinostat disrupts HIV-1 latency in patients on antiretroviral therapy | NATURE | 824 | 2012 |
| 26 | Effect of Early versus Deferred Antiretroviral Therapy for HIV on Survival | NEW ENGLAND JOURNAL OF MEDICINE | 820 | 2009 |
| 27 | Immune control of HIV-1 after early treatment of acute infection | NATURE | 796 | 2000 |
| 28 | Efavirenz plus zidovudine and lamivudine, efavirenz plus indinavir, and indinavir plus zidovudine and lamivudine in the treatment of HIV-1 infection in adults | NEW ENGLAND JOURNAL OF MEDICINE | 795 | 1999 |
| 29 | MULTIFACTORIAL NATURE OF HUMAN-IMMUNODEFICIENCY-VIRUS DISEASE - IMPLICATIONS FOR THERAPY | SCIENCE | 790 | 1993 |
| 30 | Clinical progression and virological failure on highly active antiretroviral therapy in HIV-1 patients: a prospective cohort study | LANCET | 790 | 1999 |
| 31 | Cardiovascular disease risk factors in HIV patients - association with antiretroviral therapy. Results from the DAD study | AIDS | 785 | 2003 |
| 32 | MOLECULAR TARGETS FOR AIDS THERAPY | SCIENCE | 782 | 1990 |
| 33 | Antiretroviral treatment of adult HIV infection - 2008 recommendations of the International AIDS Society USA panel | JAMA-JOURNAL OF THE AMERICAN MEDICAL ASSOCIATION | 776 | 2008 |
| 34 | A trial comparing nucleoside monotherapy with combination therapy in HIV-infected adults with CD4 cell counts from 200 to 500 per cubic millimeter | NEW ENGLAND JOURNAL OF MEDICINE | 761 | 1996 |
| 35 | Antiretroviral therapy for HIV infection in 1997 - Updated recommendations of the International AIDS Society USA panel | JAMA-JOURNAL OF THE AMERICAN MEDICAL ASSOCIATION | 745 | 1997 |
| 36 | Combination antiretroviral strategies for the treatment of pregnant HIV-1-infected women and prevention of perinatal HIV-1 transmission | JOURNAL OF ACQUIRED IMMUNE DEFICIENCY SYNDROMES | 718 | 2002 |
| 37 | Sexual Activity Without Condoms and Risk of HIV Transmission in Serodifferent Couples When the HIV-Positive Partner Is Using Suppressive Antiretroviral Therapy | JAMA-JOURNAL OF THE AMERICAN MEDICAL ASSOCIATION | 700 | 2016 |
| 38 | Post-Treatment HIV-1 Controllers with a Long-Term Virological Remission after the Interruption of Early Initiated Antiretroviral Therapy ANRS VISCONTI Study | PLOS PATHOGENS | 699 | 2013 |
| 39 | Non-adherence to highly active antiretroviral therapy predicts progression to AIDS | AIDS | 698 | 2001 |
| 40 | Sexual transmission of HIV according to viral load and antiretroviral therapy: systematic review and meta-analysis | AIDS | 697 | 2009 |
| 41 | Hepatotoxicity associated with antiretroviral therapy in adults infected with human immunodeficiency virus and the role of hepatitis C or B virus infection | JAMA-JOURNAL OF THE AMERICAN MEDICAL ASSOCIATION | 687 | 2000 |
| 42 | Heterosexual HIV-1 transmission after initiation of antiretroviral therapy: a prospective cohort analysis | LANCET | 684 | 2010 |
| 43 | Quantifying residual HIV-1 replication in patients receiving combination antiretroviral therapy | NEW ENGLAND JOURNAL OF MEDICINE | 684 | 1999 |
| 44 | Antiretroviral Treatment of Adult HIV Infection 2010 Recommendations of the International AIDS Society-USA Panel | JAMA-JOURNAL OF THE AMERICAN MEDICAL ASSOCIATION | 684 | 2010 |
| 45 | Efavirenz plasma levels can predict treatment failure and central nervous system side effects in HIV-1-infected patients | AIDS | 683 | 2001 |
| 46 | Reduction of concentration of HIV-1, in semen after treatment of urethritis: Implications for prevention of sexual transmission of HIV-1 | LANCET | 682 | 1997 |
| 47 | Improved survival among HIV-infected individuals following initiation of antiretroviral therapy | JAMA-JOURNAL OF THE AMERICAN MEDICAL ASSOCIATION | 676 | 1998 |
| 48 | Clinical progression, survival, and immune recovery during antiretroviral therapy in patients with HIV-1 and hepatitis C virus coinfection: the Swiss HIV Cohort Study | LANCET | 674 | 2000 |
| 49 | Antiretroviral Treatment of Adult HIV Infection 2012 Recommendations of the International Antiviral Society-USA Panel | JAMA-JOURNAL OF THE AMERICAN MEDICAL ASSOCIATION | 657 | 2012 |
| 50 | Treatment for adult HIV infection - 2006 recommendations of the International AIDS Society-USA panel | JAMA-JOURNAL OF THE AMERICAN MEDICAL ASSOCIATION | 654 | 2006 |
| 51 | THE SAFETY AND EFFICACY OF ZIDOVUDINE (AZT) IN THE TREATMENT OF SUBJECTS WITH MILDLY SYMPTOMATIC HUMAN-IMMUNODEFICIENCY-VIRUS TYPE-1 (HIV) INFECTION - A DOUBLE-BLIND, PLACEBO-CONTROLLED TRIAL | ANNALS OF INTERNAL MEDICINE | 651 | 1990 |
| 52 | T cell activation is associated with lower CD4(+) T cell gains in human immunodeficiency virus-infected patients with sustained viral suppression during antiretroviral therapy | JOURNAL OF INFECTIOUS DISEASES | 647 | 2003 |
| 53 | Antiretroviral therapy for HIV infection in 1998 - Updated recommendations of the International AIDS Society USA panel | JAMA-JOURNAL OF THE AMERICAN MEDICAL ASSOCIATION | 644 | 1998 |
| 54 | Severe CD4(+) T-cell depletion in gut lymphoid tissue during primary human immunodeficiency virus type 1 infection and substantial delay in restoration following highly active antiretroviral therapy | JOURNAL OF VIROLOGY | 636 | 2003 |
| 55 | Cancer risk in the swiss HIV cohort study: Associations with immunodeficiency, smoking, and highly active antiretroviral therapy | JNCI-JOURNAL OF THE NATIONAL CANCER INSTITUTE | 616 | 2005 |
| 56 | Treatment of human immunodeficiency virus infection with saquinavir, zidovudine, and zalcitabine | NEW ENGLAND JOURNAL OF MEDICINE | 606 | 1996 |
| 57 | HIV-1 and T cell dynamics after interruption of highly active antiretroviral therapy (HAART) in patients with a history of sustained viral suppression | PROCEEDINGS OF THE NATIONAL ACADEMY OF SCIENCES OF THE UNITED STATES OF AMERICA | 604 | 1999 |
| 58 | Maternal viral load, zidovudine treatment, and the risk of transmission of human immunodeficiency virus type 1 from mother to infant | NEW ENGLAND JOURNAL OF MEDICINE | 602 | 1996 |
| 59 | Biphasic kinetics of peripheral blood T cells after triple combination therapy in HIV-1 infection: A composite of redistribution and proliferation | NATURE MEDICINE | 600 | 1998 |
| 60 | Antiretroviral treatment for adult HIV infection in 2002 - Updated recommendations of the international AIDS Society-USA panel | JAMA-JOURNAL OF THE AMERICAN MEDICAL ASSOCIATION | 591 | 2002 |
| 61 | Drug-resistance genotyping in HIV-1 therapy: the VIRADAPT randomised controlled trial | LANCET | 588 | 1999 |
| 62 | Association of highly active antiretroviral therapy coverage, population viral load, and yearly new HIV diagnoses in British Columbia, Canada: a population-based study | LANCET | 588 | 2010 |
| 63 | Effectiveness of potent antiretroviral therapy on time to AIDS and death in men with known HIV infection duration | JAMA-JOURNAL OF THE AMERICAN MEDICAL ASSOCIATION | 579 | 1998 |
| 64 | Antiretroviral therapy and the prevalence and incidence of diabetes mellitus in the Multicenter AIDS Cohort Study | ARCHIVES OF INTERNAL MEDICINE | 562 | 2005 |
| 65 | Raltegravir with optimized background therapy for resistant HIV-1 infection | NEW ENGLAND JOURNAL OF MEDICINE | 557 | 2008 |
| 66 | STRATEGIES FOR ANTIVIRAL THERAPY IN AIDS | NATURE | 555 | 1987 |
| 67 | Retention in HIV Care between Testing and Treatment in Sub-Saharan Africa: A Systematic Review | PLOS MEDICINE | 553 | 2011 |
| 68 | Timing of initiation of antiretroviral therapy in AIDS-free HIV-1-infected patients: a collaborative analysis of 18 HIV cohort studies | LANCET | 553 | 2009 |
| 69 | Class-sparing regimens for initial treatment of HIV-1 infection | NEW ENGLAND JOURNAL OF MEDICINE | 546 | 2008 |
| 70 | Antiretroviral therapy for HIV infection in 1996 - Recommendations of an international panel | JAMA-JOURNAL OF THE AMERICAN MEDICAL ASSOCIATION | 544 | 1996 |
| 71 | Impact of HIV-related stigma on treatment adherence: systematic review and meta-synthesis | JOURNAL OF THE INTERNATIONAL AIDS SOCIETY | 541 | 2013 |
| 72 | Dolutegravir plus Abacavir-Lamivudine for the Treatment of HIV-1 Infection | NEW ENGLAND JOURNAL OF MEDICINE | 541 | 2013 |
| 73 | Virologic and immunologic consequences of discontinuing combination antiretroviral-drug therapy in HIV-infected patients with detectable viremia. | NEW ENGLAND JOURNAL OF MEDICINE | 540 | 2001 |
| 74 | NEVIRAPINE RESISTANCE MUTATIONS OF HUMAN-IMMUNODEFICIENCY-VIRUS TYPE-1 SELECTED DURING THERAPY | JOURNAL OF VIROLOGY | 533 | 1994 |
| 75 | Human immunodeficiency virus controllers: Mechanisms of durable virus control in the absence of antiretroviral therapy | IMMUNITY | 531 | 2007 |
| 76 | Response to antiretroviral treatment in HIV-1-infected individuals with allelic variants of the multidrug resistance transporter 1: a pharmacogenetics study | LANCET | 531 | 2002 |
| 77 | Persistence of HIV-1 transcription in peripheral-blood mononuclear cells in patients receiving potent antiretroviral therapy | NEW ENGLAND JOURNAL OF MEDICINE | 529 | 1999 |
| 78 | Impact of new antiretroviral combination therapies in HIV infected patients in Switzerland: prospective multicentre study | BMJ-BRITISH MEDICAL JOURNAL | 524 | 1997 |
| 79 | Epidemiology of human immunodeficiency virus-associated opportunistic infect-ions in the United States in the era of highly active antiretroviral therapy | CLINICAL INFECTIOUS DISEASES | 517 | 2000 |
| 80 | Patterns, correlates, and barriers to medication adherence among persons prescribed new treatments for HIV disease | HEALTH PSYCHOLOGY | 513 | 2000 |
| 81 | Lopinavir-ritonavir versus nelfinavir for the initial treatment of HIV infection | NEW ENGLAND JOURNAL OF MEDICINE | 513 | 2002 |
| 82 | DESIGNING CD4 IMMUNOADHESINS FOR AIDS THERAPY | NATURE | 507 | 1989 |
| 83 | Highly active antiretroviral therapy and incidence of cancer in human immunodeficiency virus-infected adults | JNCI-JOURNAL OF THE NATIONAL CANCER INSTITUTE | 504 | 2000 |
| 84 | Safety and efficacy of raltegravir-based versus efavirenz-based combination therapy in treatment-naive patients with HIV-1 infection: a multicentre, double-blind randomised controlled trial | LANCET | 496 | 2009 |
| 85 | Relationship between T cell activation and CD4(+) T cell count in HIV-seropositive individuals with undetectable plasma HIV RNA levels in the absence of therapy | JOURNAL OF INFECTIOUS DISEASES | 494 | 2008 |
| 86 | Discovery of Raltegravir, a potent, selective orally bioavailable HIV-integrase inhibitor for the treatment of HIV-AIDS infection | JOURNAL OF MEDICINAL CHEMISTRY | 492 | 2008 |
| 87 | The WHO public-health approach to antiretroviral treatment against HIV in resource-limited settings | LANCET | 488 | 2006 |
| 88 | Survival of HIV-positive patients starting antiretroviral therapy between 1996 and 2013: a collaborative analysis of cohort studies | LANCET HIV | 486 | 2017 |
| 89 | First human trial of a DNA-based vaccine for treatment of human immunodeficiency virus type 1 infection: Safety and host response | JOURNAL OF INFECTIOUS DISEASES | 484 | 1998 |
| 90 | Kinetics of response in lymphoid tissues to antiretroviral therapy of HIV-1 infection | SCIENCE | 483 | 1997 |
| 91 | The cytochrome P4502B6 (CYP2B6) is the main catalyst of efavirenz primary and secondary metabolism: Implication for HIV/AIDS therapy and utility of efavirenz as a substrate marker of CYP2B6 catalytic activity | JOURNAL OF PHARMACOLOGY AND EXPERIMENTAL THERAPEUTICS | 482 | 2003 |
| 92 | HIV infection induces changes in CD4(+) T-cell phenotype and depletions within the CD4(+) T-cell repertoire that are not immediately restored by antiviral or immune-based therapies | NATURE MEDICINE | 473 | 1997 |
| 93 | Immune reconstitution inflammatory syndrome in patients starting antiretroviral therapy for HIV infection: a systematic review and meta-analysis | LANCET INFECTIOUS DISEASES | 471 | 2010 |
| 94 | Depression and HIV/AIDS Treatment Nonadherence: A Review and Meta-analysis | JAIDS-JOURNAL OF ACQUIRED IMMUNE DEFICIENCY SYNDROMES | 465 | 2011 |
| 95 | A syndrome of lipoatrophy, lactic acidaemia and liver dysfunction associated with HIV nucleoside analogue therapy: contribution to protease inhibitor-related lipodystrophy syndrome | AIDS | 464 | 2000 |
| 96 | Self-report measures of antiretroviral therapy adherence: A review with recommendations for HIV research and clinical management | AIDS AND BEHAVIOR | 463 | 2006 |
| 97 | Long-lasting recovery in CD4 T-cell function and viral-load reduction after highly active antiretroviral therapy in advanced HIV-1 disease | LANCET | 460 | 1998 |
| 98 | Antiretroviral therapy adherence and viral suppression in HIV-infected drug users: Comparison of self-report and electronic monitoring | CLINICAL INFECTIOUS DISEASES | 453 | 2001 |
| 99 | Cost-effectiveness of screening for HIV in the era of highly active antiretroviral therapy | NEW ENGLAND JOURNAL OF MEDICINE | 453 | 2005 |
| 100 | TREATMENT WITH LAMIVUDINE, ZIDOVUDINE, OR BOTH IN HIV-POSITIVE PATIENTS WITH 200 TO 500 CD4+ CELLS PER CUBIC MILLIMETER | NEW ENGLAND JOURNAL OF MEDICINE | 451 | 1995 |

**Supplementary Figure 1 Cooperation of different institutions**

**
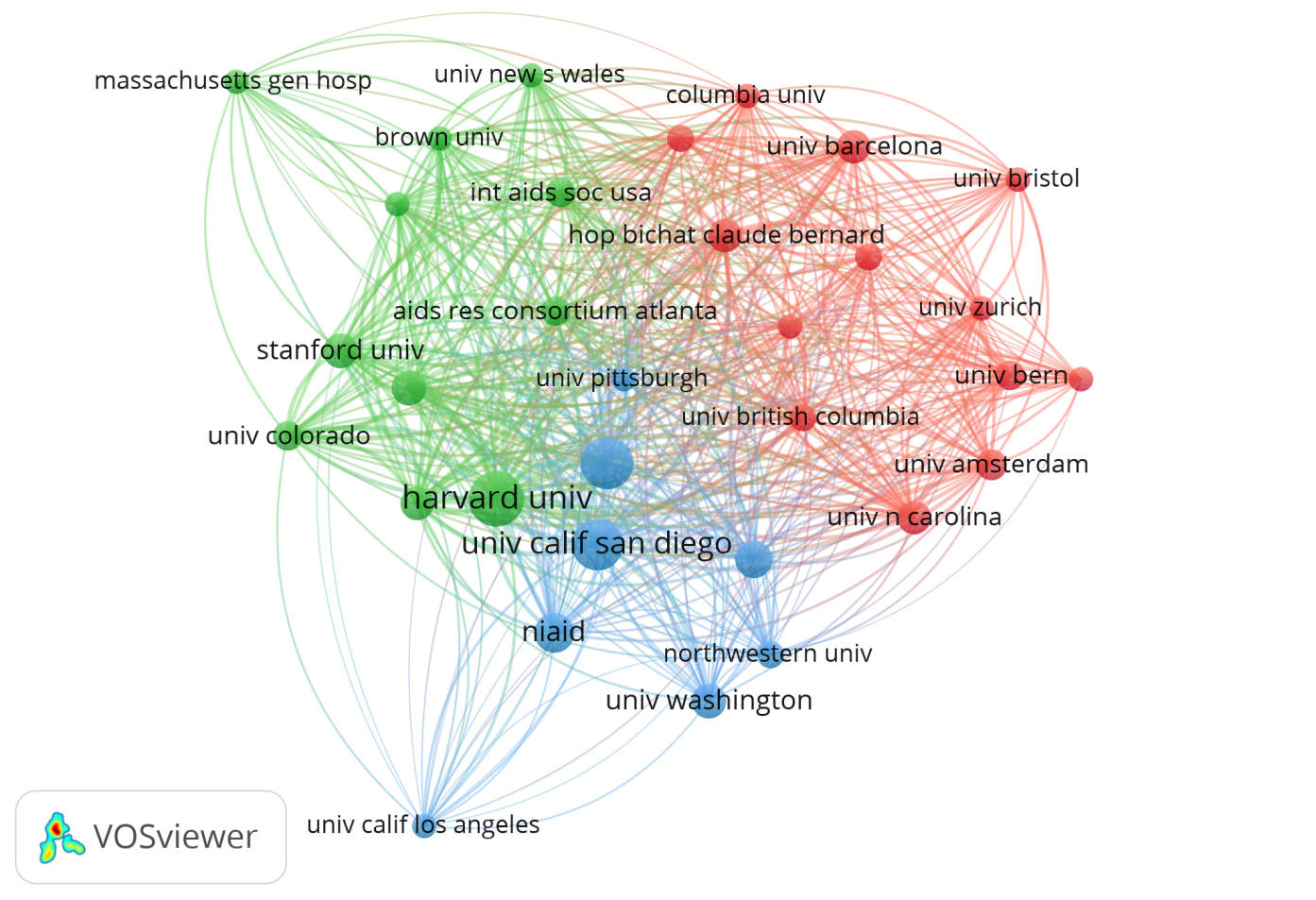
**

**Supplementary Figure 2 Cooperation of different authors**


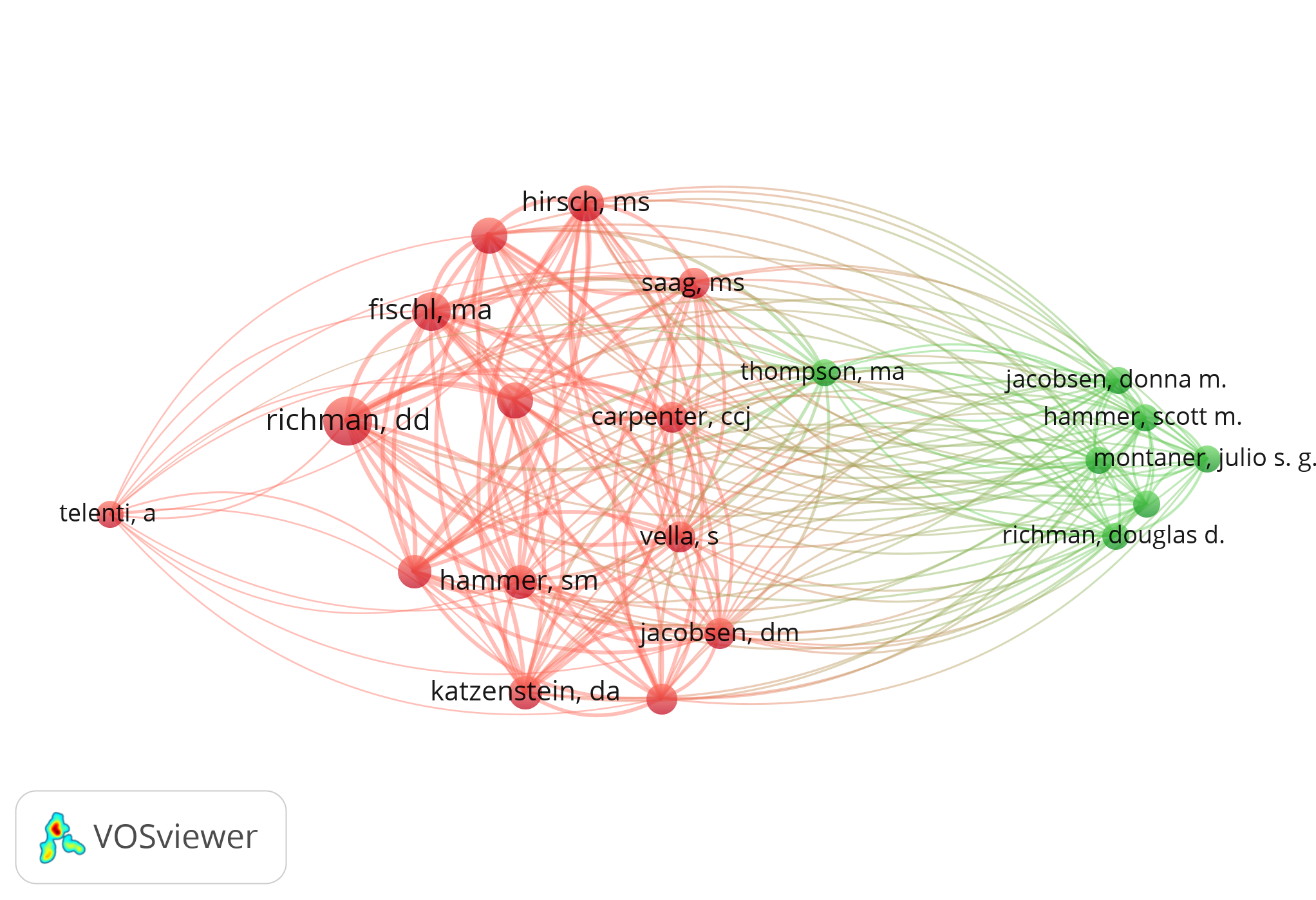

Supplement: Supplementary file 1 [file Table1.DOCX]
